# RNF8–CDH1 Co-Expression Predicts Clinical Benefit of Chemoradiotherapy in Triple-Negative Breast Cancer

**DOI:** 10.3390/jpm11070655

**Published:** 2021-07-13

**Authors:** Chieh-Ni Kao, Sin-Hua Moi, Ming-Feng Hou, Chi-Wen Luo, Fang-Ming Chen, Mei-Ren Pan

**Affiliations:** 1 Graduate Institute of Clinical Medicine, Kaohsiung Medical University, Kaohsiung 807, Taiwan; jennykao0320@gmail.com; 2 Division of Breast Oncology and Surgery, Department of Surgery, Kaohsiung Medical University Hospital, Kaohsiung 807, Taiwan; cwlo0623@gmail.com (C.-W.L.); fmc5464@gmail.com (F.-M.C.); 3 Center of Cancer Program Development, E-Da Cancer Hospital, I-Shou University, Kaohsiung 824, Taiwan; moi9009@gmail.com; 4 Drug Development and Value Creation Research Center, Kaohsiung Medical University, Kaohsiung 807, Taiwan

**Keywords:** RNF8, CDH1, TNBC, chemoradiotherapy

## Abstract

Triple-negative breast cancer (TNBC) is the most aggressive breast cancer subtype and exhibits an overall poor outcome. Due to the lack of targeted therapy, conventional systemic chemotherapy has been the main strategy for the treatment of TNBC. Further evidence has shown that combining radiation with chemotherapy is also a suitable treatment based on DNA repair deficiencies in patients with TNBC. However, the preferred treatment for metastatic TNBC remains unclear. Therefore, identification of biomarkers is an unmet need in personalized therapy for TNBC. RNF8 (ring finger protein 8) is a ubiquitin ligase implicated in TNBC metastasis; however, its role in TNBC pathogenesis is unclear. The purpose of the present study was to investigate the roles of the RNF8–CDH1(Cadherin 1) axis in node-positive TNBC patients. We found that the *RNF8^high^*/*CDH1^low^* index was significantly higher in patients with TNBC than in patients without TNBC. Furthermore, patients with an *RNF8^high^*/*CDH1^low^* index displayed poorer outcomes than those with an *RNF8^low^*^-medium^/*CDH1^medium^*^-high^ index. Notably, as compared to patients with an *RNF8^low^*^-medium^/*CDH1^medium^*^-high^ index, those with an *RNF8^high^*/*CDH1^low^* index had a poorer survival rate with chemotherapy treatment alone. The combination of radiation and chemotherapy resulted in a better survival rate than chemotherapy alone in patients with an *RNF8^high^*/*CDH1^low^* index. Taken together, the *RNF8^high^*/*CDH1^low^* index not only functions as a prognostic and therapeutic marker but may also act as a target in the development of anti-cancer agents for patients with TNBC.

## 1. Introduction

Triple-negative breast cancer (TNBC) is a breast cancer subtype that lacks an estrogen receptor (ER), a progesterone receptor (PR), and a human epidermal growth factor receptor 2 (HER2). TNBCs account for 10–20% of breast cancer cases and are associated with a high level of metastases, high recurrence rates, and the worst prognoses [1]. Approximately 90% of cancer-related deaths are caused by metastatic diseases [2]. Sometimes, patients with TNBC do not have a second chance to fight the disease owing to its aggressive progression. According to the National Comprehensive Cancer Network (NCCN) v5.2021 guidelines and ESO-ESMO guidelines 2018, chemotherapy is still a standard option for the treatment of metastatic TNBC (mTNBC) [3,4]. Recently, evidence further indicated that a combination of atezolizumab and nab-paclitaxel might be used in patients with programmed death-ligand 1 (PD-L1)-positive advanced TNBC patients for prolonged progression-free survival [5]. However, the preferred treatment for metastatic TNBC with visceral crisis remains controversial and requires further study. Thus, obtaining the most accurate treatment is critical. Therefore, it is important to understand the mechanisms of metastasis to improve the therapeutic outcomes of TNBC.

Epithelial–mesenchymal transition (EMT) is a crucial step in the process of invasion and metastasis in solid tumors [6]. EMT can be induced or regulated by signaling pathways controlled by various growth factors and cytokines, including TGF-β, VEGF, IL6, and EGF, as well as the tyrosine kinase receptor pathway [7,8,9,10]. Activation of these pathways leads to transcriptional repression of a series of cell adhesion molecules, including E-cadherin, also known as CDH1(cadherin 1), to further drive cellular mobility [11]. Studies on TNBC cells and tissues have shown that they commonly exhibited an activated EMT program and possessed an enriched cancer stem cell (CSC) population [12,13]. Metastatic and stem-ness-like tumors are generally refractory to primary tumors, with poor response rates and shortened overall survival. Therefore, exploring the molecular basis underlying the status of CSCs and EMT in TNBC may stratify the prognostic and possible therapeutic classifications.

A prior analysis of patient samples further identified that the CSCs accounting for TNBC metastasis harbored an EMT gene signature including *TWIST* upregulation [14,15]. Expression of *TWIST* is repressed in normal adult tissues but upregulated in TNBC and high-grade breast cancer. TWIST is a well-known transcriptional factor that regulates various physiological processes, including EMT, tumor growth, and chemoresistance [15,16,17]. A well-known example is that TWIST is a transcriptional repressor of CDH1 gene expression in contributing a malignant phenotype of breast cancer [18]. It has been reported that TGFβ, hypoxia, STAT3, and RNF8 (ring finger protein 8) triggered the expression of TWIST underlying transcriptional and post-translational regulation [19,20,21,22]. Therefore, verifying the roles of TWIST-mediated downstream genes and targeting upstream regulators of TWIST may provide a potential strategy for the development of prognostic and therapeutic tools for TNBC.

RNF8, an E3 ubiquitin ligase, is a well-known critical DNA damage-responsive protein involved in the process of DNA repair [23,24,25]. Recently, several lines further demonstrated that RNF8 played a role in inducing EMT and metastasis in TNBC [22]. Notably, RNF8 promotes EMT via K63-linked ubiquitination and subsequent activation and stabilization of the TWIST protein to further control TWIST-mediated downstream gene expression. This study aimed to further clarify the roles of RNF8 in clinical patient outcomes. To this end, we conducted a serial comprehensive analysis including RNA sequencing (RNA-seq) data from The Cancer Genome Atlas Breast Invasive Carcinoma (TCGA-BRCA) to address the role of RNF8- and TWIST-mediated downstream genes in patients with lymph node (LN) invasion using the Genomic Data Commons (GDC) data portal. In addition to investigating RNF8’s role in clinicopathological features and the patient survival rate, we aimed to evaluate its value in therapeutic strategies for patients with TNBC.

## 2. Materials and Methods

### 2.1. Data Source

All data were downloaded from cBioPortal (https://www.cbioportal.org/, accessed date 10 January 2021). The baseline, tumor, and treatment characteristics, and RNA-seq expression in the study population were extracted from the Breast Invasive Carcinoma of the Cancer Genome Atlas Program PanCancer database (TCGA-BRCA). The baseline characteristics included age, race, ethnicity, and breast cancer subtypes. The tumor characteristics included tumor staging (AJCC), lymph node staging (AJCC), and pathological stage. The treatment characteristics included surgery, radiation therapy, chemotherapy, hormone therapy, and target therapy. The study population was divided into the LN subgroup according to LN staging. Patients with LN staging of NX and N0 were defined as the LN-negative (LN−) subgroup, and patients with LN staging of N1 to N3 were defined as the LN-positive (LN+) subgroup. Disease-free survival (DFS) was considered as the primary endpoint. Patients who had progression or recurrence events during the follow-up interval were considered as progressive disease; otherwise, they were considered disease-free. Overall survival (OS) was considered as a secondary endpoint and was defined by the all-cause mortality status of the study population.

### 2.2. RNA-seq Expression

The gene expression profile was experimentally determined using the Illumina HiSeq 2000 RNA Sequencing platform at the University of North Carolina TCGA genome characterization center. RNA-seq expression was computed using the mean normalized (per gene) gene expression by RNA-seq across all TCGA cohorts. In this study, RNA-seq expression of *RNF8* and related genes was reported in reads per kilobase million (RPKM) and shown in the log2 (norm_count + 1)-transformed RNA-Seq by expectation-maximization (RSEM) pan-cancer normalized count.

### 2.3. UALCAN Data Analysis

UALCAN (http://ualcan.path.uab.edu, accessed date 19 June 2021) is a web resource that explores cancer transcriptome data, including RNA-seq, promoter methylation, correlation, and survival, using TCGA data. *RNF8* expression under different subtypes was analyzed in patients with breast cancer using UALCAN. A *p*-value of less than 0.05 (*p* < 0.05) was considered statistically significant for all results.

### 2.4. Statistical Analyses

The baseline characteristics of the study population were summarized as frequency and percentages or median and range, and the difference in distribution between the LN subgroups was estimated using an independent two-sample *t*-test, a chi-square test, or Fisher’s exact test. The correlation of RNA-seq expression between *RNF8* and related genes was estimated using Pearson’s correlation analysis. The correlation matrix of *RNF8* and related genes was illustrated using a heatmap. *RNF8* significantly correlated genes were considered candidate genes (GENE). RNA-seq expression of *RNF8* and GENE in the LN and TNBC subgroups was visualized using boxplots, and the difference in RNA-seq expression was tested by the Wilcoxon rank-sum test. Optimal cut-off values for RNA-seq expression of *RNF8* and each GENE were determined using the X-tile algorithm according to DFS [26]. *RNF8* and all GENE were divided into high-, medium-, and low-RNA-seq expression strata according to the corresponding optimal cut-off points. The distribution of the study population according to the *RNF8* strata and RNA-seq expression strata of each GENE was illustrated using faceted bar charts in order to identify the differences in TNBC and non-TNBC proportions in each *RNF8* × GENE stratum. The survival curve of RNA-seq expression strata in *RNF8*, GENE, and *RNF8* × GENE was estimated using the Kaplan–Meier estimator, and the survival difference between strata was tested using the log-rank test. All *p*-values were two-sided, and statistical significance was set at *p* < 0.05. All statistical analyses were performed using the computing environment R 4.0.3 (R Core Team, 2020).

## 3. Results

### 3.1. Clinicopathological Characteristics and Progression of Breast Cancer

The clinicopathological characteristics of 498 patients with breast cancer were collected and summarized from TCGA-BRCA of the GDC data portal. The presence of LN metastasis is essential for the formulation of treatment strategies for breast cancer. To build a nomogram that predicts LN metastasis in patients with breast cancer, the obtained breast cancer samples were further divided into LN-negative (LN−, *n* = 234) and LN-positive (LN+, *n* = 264) groups. A total of 234 LN− patients, with a median age of 57 years, and 264 LN+ patients, with a median age of 56 years, were analyzed. Overall, several clinicopathological factors, including subtypes (*p* = 0.002), T staging (*p* < 0.001), N staging (*p* < 0.001), pathological stage (*p* < 0.001), and treatment choices including radiation therapy and chemotherapy (*p* < 0.001), were significantly different between LN− and LN+ patients (Table 1).

### 3.2. A Significant Correlation among RNF8, SNAI1, and CDH1 mRNA Expression in Patients with Breast Cancer

It is well known that RNF8 is a RING finger E3 ligase involved in DNA damage repair. A prior study demonstrated that RNF8 facilitated chemoresistance and EMT through ubiquitination of TWIST [22]. TWIST is a basic helix-loop-helix transcription factor required for the activation of oncogenic and mesenchymal genes, including *AKT2, TGFB2, PDGFRA, CDH1, SNAI1, CLDN1, RKIP, NPHS2*, and *CDH2* [27,28,29,30,31,32]. To further verify the correlation of *RNF8*–*TWIST*-mediated downstream genes in breast cancer, Pearson’s correlation analysis first confirmed that positive correlations existed between *RNF8* and upregulated EMT markers. *SNAI1* showed a significant positive correlation (*r* value = 0.185). *CDH1* was negatively correlated with *RNF8* (*r* value = −0.162) (Table 2).

### 3.3. High Levels of RNF8 Are Present in Patients with TNBC

**Figure 1:**
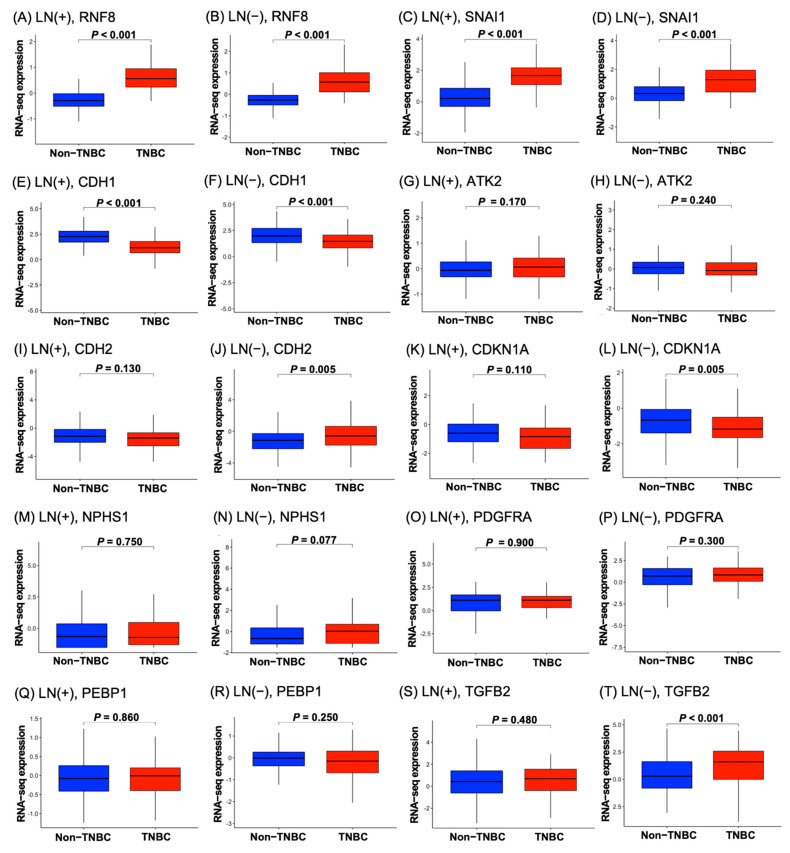
Boxplot of RNA-seq expression in TNBC and non-TNBC according to LN invasion status. (**A**,**B**) *RNF8*, (**C**,**D**) *SNAI1*, (**E**,**F**) *CDH1*, (**G**,**H**) *AKT2*, (**I**,**J**) *CDH2*, (**K**,**L**) *CDKN1A*, (**M**,**N**) *NPHS1*, (**O**,**P**) *PDGFRA*, (**Q**,**R**) *PEBP1*, and (**S**,**T**) *TGFB2*.

**Figure 2:**
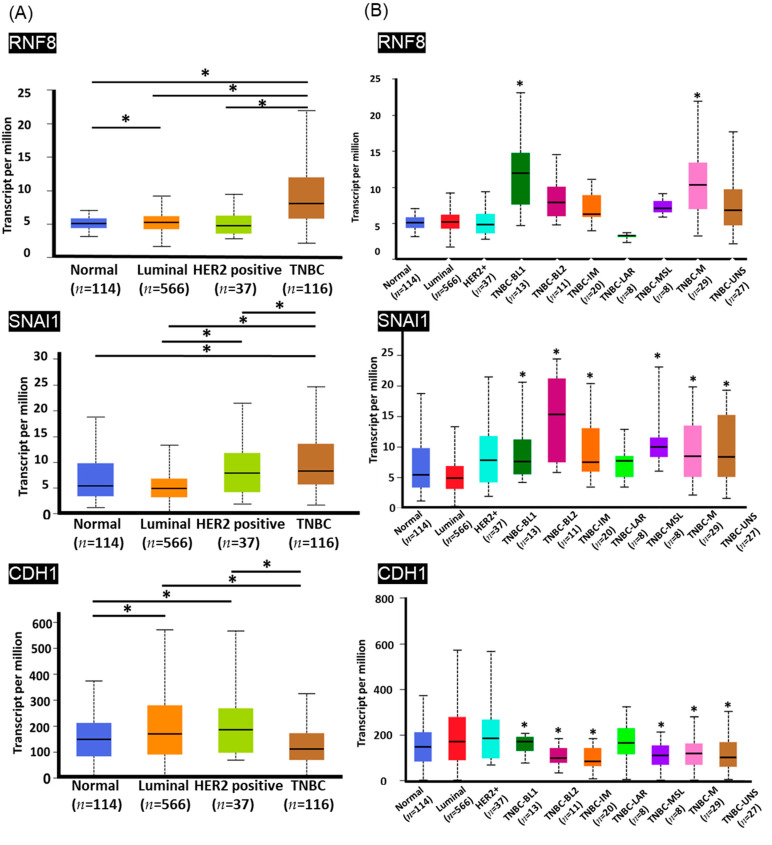
UALCAN portal analysis of breast cancer samples from the TCGA database. (**A**) Comparison of *RNF8*, *SNAI1*, and *CDH1* expression levels between normal and different subtypes of breast cancer samples. Normal, *n* = 114; luminal, *n* = 566; HER2+, *n* = 37; TNBC, *n* = 116. (**B**) Expression of RNF8, *SNAI1*, and *CDH1* between different TNBC subgroups. Normal, *n* = 114; luminal, *n* = 566; HER2+, *n* = 37; TNBC-BL1, *n* = 13; TNBC-BL2, *n* = 11; TNBC-IM, *n* = 20; TNBC-LAR, *n* = 8; TNBC-MSL, *n* = 8; TNBC-M, *n* = 29; TNBC-UNS, *n* = 27. * *p* < 0.05. TNBC, triple-negative breast cancer; TCGA, The Cancer Genome Atlas.

Breast cancer is a heterogeneous disease that is represented by different molecular subtypes and displays various patterns of gene expression, clinical features, responses to treatment, and prognosis. To investigate the contribution of *RNF8* to cancer progression in different breast cancer subtypes, we determined *RNF8*, *SNAI1*, and *CDH1* expression levels in TNBC and non-TNBC breast cancer subtypes using the TCGA RNA-seq dataset. As it is shown in Figure 1, an increase in *RNF8* and *SNAI1* mRNA levels and a decrease in *CDH1* mRNA were observed in LN+ and LN- TNBC patients. In contrast to *RNF8*, *SNAI1*, and *CDH1* mRNA, other *TWIST*-related downstream genes, including *AKT2, TGFB2, PDGFRA, RKIP, NPHS2*, and *CDH2*, were not altered between patients with and without TNBC. These data indicate that *RNF8*, *SNAI1*, and *CDH1* might play a role in TNBC. Therefore, we focused on studying the correlation between *RNF8*, *SNAI1*, and *CDH1* in patients with TNBC. To further verify the roles of *RNF8* in TNBC, we analyzed its expression in different TNBC subgroups using the UALCAN database. Consistent with the findings shown in Figure 1, high levels of *RNF8*, *SNAI1*, and *CDH1* were observed in TNBC compared with other subtypes of breast cancer (Figure 2A). According to gene expression profiling and treatment response, TNBC is classified into six distinct subgroups: basal-like 1 (BL1), basal-like 2 (BL2), mesenchymal (M), mesenchymal stem-like (MSL), immunomodulatory (IM), and luminal androgen receptor (LAR) [33]. Notably, *RNF8* was significantly expressed in the BL1 and M subgroups of TNBC; *SNAI1* was significantly expressed in the BL2, IM, MSL, M, and UNS subgroups of TNBC, compared to the luminal and HER2+ subtypes; and *CDH1* was significantly downregulated in the BL2, MSL, M, and UNS subgroups of TNBC, compared to BL1 (Figure 2B). Notably, evidence indicates that the BL1 subtype is characterized by the enrichment of cellular proliferation and the DNA damage response. The M subtype is specifically enriched in the components of differentiation, migration, and tumor growth. This evidence demonstrates that targeting *RNF8* might provide therapeutic strategies for the blockade of metastasis and DNA repair ability.

### 3.4. Patients with TNBC Display RNF8^high^/CDH1^low^ Expression

**Figure 3:**
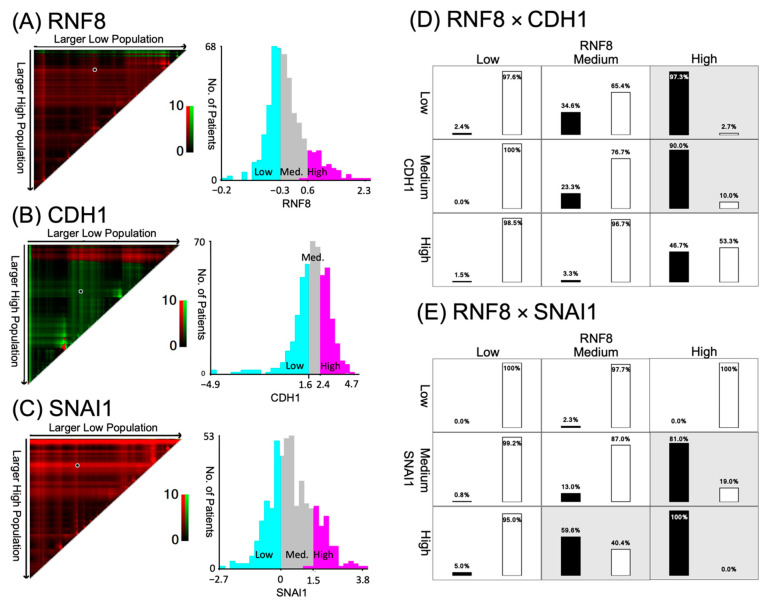
The high, medium, and low subgroups for RNA-seq expression according to DFS using X-tile algorithm. (**A**) *RNF8*, (**B**) *CDH1*, and (**C**) *SNAI1*. The right panel graphically represents a right-triangular grid in which each point indicates the RNA-seq expression in a study population. The x-axis represents all possible “low” samples with the size of the low samples increasing from left to right, and the y-axis represents all possible “high” samples with the size of the high samples increasing from top to bottom. The left panel represents the number of patients in high, medium, and low subgroups stratified by X-tile algorithm. (**D**) Distribution of TNBC and non-TNBC in each *RNF8* × *CDH1* strata among LN- subgroup. (**E**) Distribution of TNBC and non-TNBC in each RNF8×SNAI1 strata among LN+ subgroup. Black bar represents proportion of TNBC; white bar represents proportion of non-TNBC. Stratum marked in gray color indicates higher TNBC proportion compared to non-TNBC. DFS, disease-free survival; TNBC, triple-negative breast cancer.

Next, we used X-tile analysis to establish the cut-off values of *RNF8*, *SNAI1*, and *CDH1* expression in the TCGA database and survival analyses (Figure 3A–C). The X-tile analysis with the optimal cut-off value of *RNF8* was categorized as low (−0.2–−0.3), intermediate (−0.3–0.6), and high (0.6–2.3). *CDH1* was categorized as low (−4.9–1.6), intermediate (1.6–2.4), and high (2.4–4.7). *SNAI1* was categorized as low (−2.7–0), intermediate (0–1.5), and high (1.5–3.8) based on DFS information. The analyses showed that *RNF8^high^*/*CDH1^low^* and *RNF8^high^*/*SNAI1*^high^ expression was positively associated in patients with TNBC subtypes, compared to non-TNBC subtypes (Figure 3D,E). These data suggest that the patterns of *RNF8^high^*/*CDH1^low^* and *RNF8^high^*/*SNAI1* ^high^ might function as diagnostic markers for distinguishing between TNBC and non-TNBC cells.

### 3.5. RNF8^high^/CDH1^low^ Functions as a Poor Prognostic Marker for LN+ TNBC Patients

**Figure 4:**
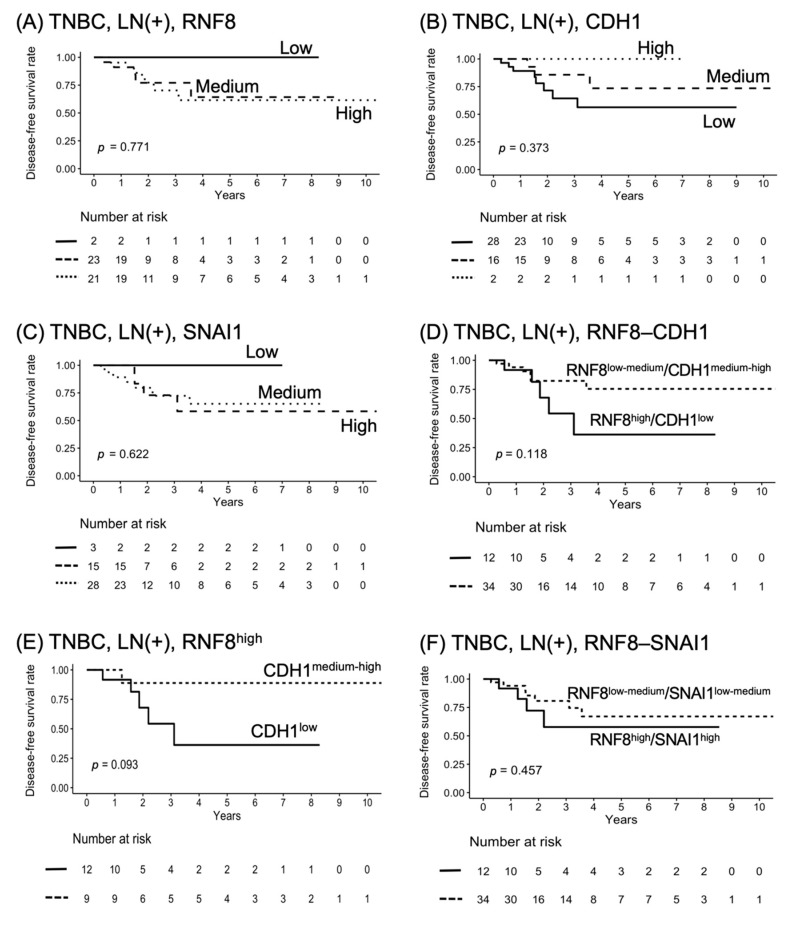
Kaplan–Meier plot for DFS in LN+ TNBC breast cancer according to high-, medium-, and low-RNA-seq expression strata of (**A**) *RNF8*, (**B**) *CDH1*, (**C**) *SNAI1*, (**D**) *RNF8* × *CDH1*, (**E**) *RNF8^high^*/*CDH1^low^* vs. *RNF8^high^*/*CDH1^medium^*^-high^, and (**F**) *RNF8* × *SNAI1* in LN+ TNBC according to RNA-seq expression. DFS, disease-free survival; LN, lymph nodes; TNBC, triple-negative breast cancer.

LN positivity is positively correlated with a higher recurrence rate of TNBC and poorer clinical outcomes. To verify the clinical role of the levels of *RNF8*, *SNAI1*, and *CDH1* in outcomes in LN+ TNBC patients, we used Kaplan–Meier survival curves to evaluate the predictive value of their expression levels on DFS. As it is shown in Figure 4A–C, there was a trend toward poorer DFS in LN+ patients with *RNF8^medium^*/^high^ and *SNAI1^medium^*/^high^. Patients with *CDH1^low^*/^medium^ had a minor trend toward poorer DFS, compared to *CDH1^high^*. This evidence indicates the levels of *RNF8^medium^*/^high^ and *SNAI1^medium^*/^high^ as high-risk strata in LN+ patients, compared to *RNF8^low^* and *SNAI1^low^*. Then, LN+ TNBC patients were clustered into two subgroups according to the expression status derived from two gene clusters. Figure 4D compares the survival curves between *RNF8^high^*/*CDH1^low^* and *RNF8^low^*^-medium^/*CDH1^medium^*^-high^ in LN+ and LN+ TNBC patients. Notably, there was a trend toward poorer DFS in LN+ patients with *RNF8^high^*/*CDH1^low^* expression than in those with *RNF8^low^*^-medium^/*CDH1^medium^*^-high^. It is worth noting that *RNF8^high^*/*CDH1^low^* showed a trend of poor DFS when compared to *RNF8^high^*/*CDH1^medium-high^*. These data suggest that the levels of *CDH1* function as a determining factor in the *RNF8*-mediated malignant phenotype in LN+ TNBC (Figure 4E). In contrast, there was no difference between *RNF8^high^*/*SNAL1^high^* and *RNF8^low^*^-medium^/*SNAL1^low^*^-medium^ in LN+ TNBC patients (Figure 4F). Taken together, these data suggest that the RNF8–CDH1 axis might be an optimal cluster in the impact of baseline clinical characteristics and risk on survival outcome.

### 3.6. Radiation Combined with Chemotherapy Improves the Survival of Node-Positive RNF8^high^/CDH1^low^

**Figure 5:**
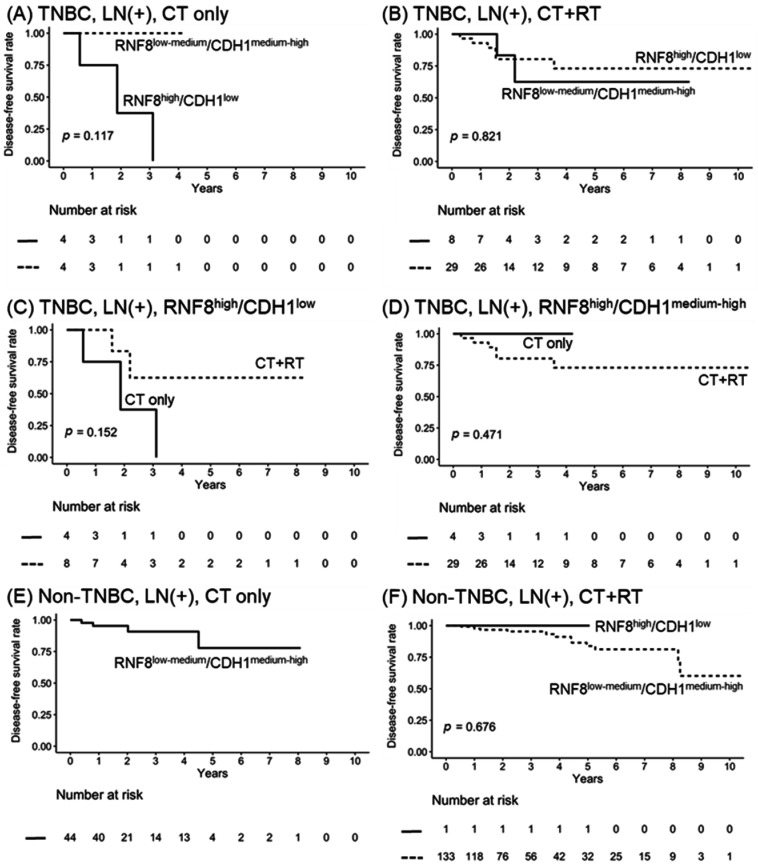
Kaplan–Meier plot for DFS in LN+ TNBC breast cancer according to high-, medium-, and low-RNA-seq expression strata of (**A**) *RNF8^high^*/*CDH1^low^* vs. *RNF8^low^*^-medium^/*CDH1^medium^*^-high^ in CT treatment, (**B**) RNF8^high^/CDH1^low^ vs. RNF8^low-medium^/CDH1^medium-high^ in CT+RT treatment, (**C**) RNF8^high^/CDH1^low^ in CT and CT + RT treatment, (**D**) *RNF8^high^*/*CDH1^medium^*^-high^ in CT and CT + RT treatment, (**E**) *RNF8^high^*/*CDH1^low^* vs. *RNF8^low^*^-medium^/ *CDH1^medium^*^-high^ in CT treatment of non-TNBC, and (**F**) *RNF8^high^*/*CDH1^low^* vs. *RNF8^low^*^-medium^/*CDH1^medium^*^-high^ in CT + RT treatment of non-TNBC RNA-seq expression. CT, chemotherapy; DFS, disease-free survival; LN, lymph nodes; RT, radiotherapy; TNBC, triple-negative breast cancer.

Evidence indicates that adjuvant radiotherapy (RT) reduces the 10-year first risk of recurrence after surgery and systemic therapy in LN+ breast cancers [34]. The NCCN guidelines indicate that patients with more than four positive LN should receive RT. In addition to this rule, identification of biomarkers to provide predictive information regarding a tumor’s intrinsic radio-response to treatment could avoid radiation-induced toxicity in patients and establish a personalized therapeutic strategy. RNF8 is a DNA damage response protein that contributes to DNA repair. To verify the role of *RNF8^high^*/*CDH1^low^* in response to RT, LN+ TNBC patients were treated with RT or non-RT, and survival outcomes were analyzed according to the expression of *RNF8^high^*/*CDH1^low^*. As it is shown in Figure 5A, *RNF8^high^*/*CDH1^low^* showed a strong trend of poor PFS when compared to *RNF8^low^*^-medium^/*CDH1^medium^*^-high^ in patients receiving chemotherapy alone. Notably, LN+ TNBC patients with *RNF8^high^*/*CDH1^low^* and *RNF8^low^*^-medium^/*CDH1^medium^*^-high^ all displayed a better response to the combination of RT treatment (Figure 5B). These data demonstrate that combined chemotherapy and radiation improves the survival of node-positive *RNF8^high^*/*CDH1^low^* (Figure 5C). In contrast, there was no difference in the survival rate in patients with *RNF8^high^*/*CDH1^medium^*^-high^ treated with CT alone, and with the CT and RT combination (Figure 5D). As it is shown in Figure 3A, only 2.7% of *RNF8^high^*/*CDH1^low^* was observed in non-TNBC patients. Therefore, there is no meaning in the evaluation of the status of *RNF8^high^*/*CDH1^low^* in non-TNBC patients regarding the survival rate (Figure 5E,F). In summary, these data indicate that the status of *RNF8^high^*/*CDH1^low^* might provide a guideline for determining combined RT therapy after surgery and systemic therapy in LN+ TNBC patients.

## 4. Discussion

Compared to other subtypes of breast cancer, TNBC has a high metastatic ability and a poor prognosis. Therefore, it is crucial to understand the mechanisms of metastasis to improve the therapeutic outcomes of TNBC. Studies have indicated that TNBC cells displayed an enriched CSC population and activated the EMT program [12,13]. TWIST overexpression has been shown to function as an EMT initiator in the development of TNBC [14,15,16,17]. These studies unequivocally emphasize the pathological importance of TWIST in TNBC, suggesting that blocking EMT by inhibiting TWIST is a compelling approach to targeting TNBC. In this study, we conducted a serial comprehensive analysis including RNA-seq data to address the correlation among *RNF8*, TWIST-mediated downstream genes, and the survival rate in patients with TNBC receiving either CT alone or CT with RT treatment. We found that *RNF8^high^* and *CDH1^low^* were significantly more common in patients with TNBC than in patients without TNBC. Patients with *RNF8^high^*/*CDH1^low^* displayed a shorter survival rate than those with *RNF8^low^*^-medium^/*CDH1^medium^*^-high^. Notably, combining RT with CT could effectively improve the survival rate in patients with *RNF8^high^*/*CDH1^low^* compared to CT alone. *RNF8^low^*^-medium^/*CDH1^medium^*^-high^ plays a role as a better diagnostic index and is also associated with a better treatment response in patients treated with CT alone.

RNF8 is a ubiquitin E3 ligase that was initially implicated in DNA damage signaling. In recent years, several studies have indicated that RNF8 promotes EMT via K63-linked ubiquitination and subsequent activation and stabilization of EMT-associated proteins, including TWIST, Slug, and β-catenin, in driving cancer metastasis [22,35,36]. Notably, our data also indicate that an increase in *RNF8* and *SNAI1* mRNA levels and a decrease in *CDH1* mRNA were observed in malignant subtypes of breast cancer, i.e., TNBC patients (Figure 1).

Recently, genetic profiling has been discovered as a strategy to personalized therapy in clinical research on TNBC treatment. For example, PARP inhibitors have been suggested for use in germline *BRCA* mutation-associated breast cancer (gBRCAm-BC), and checkpoint inhibitor atezolizumab combined with nab-paclitaxel in programmed cell death-ligand 1-positive (PD-L1+) advanced TNBC [37,38]. Although neoadjuvant therapy has focused on combinations of systemic agents to optimize the pathologically complete response, TNBC still has a poor prognosis and clinical outcomes. Generally, tumor heterogeneity is a major concern for poor treatment response in patients with TNBC. Lehman and colleagues first reported further classification of TNBC based on the tumor gene expression profile [33,39]. By analyzing microarray data, they identified six molecular subtypes characterized not only by distinct patterns of the gene expression signature but also by very diverse clinical behaviors; basal-like subtypes (BL1 and BL2) constituted approximately 70% of TN tumors. The BL1 subtype expresses genes involved in cell cycle regulation, cell proliferation, and DNA damage response, whereas BL2 highly expresses genes involved in the cell cycle, cell division, and growth factor signaling. BL1 tumors are almost exclusively of a ductal histology and a high grade and achieve a higher pathological complete response (pCR) rate when treated with neoadjuvant chemotherapy. Explicitly, BL2-subtype tumors are less likely to achieve pCR and have a higher risk of recurrence compared to BL1. For aggressive tumors such as TNBC, pCR is accepted as a favorable prognostic marker associated with long-term survival benefits [40]. Mesenchymal (M) and mesenchymal stem-like (MSL) subtypes show high expression of genes involved in EMT and growth factor signaling (EGFR, PDGFR, PI3K/mTOR, Src). Tumors in the M category are less sensitive to chemotherapy than BL1 and preferentially metastasize to the lungs. Metaplastic carcinomas are mostly of the M subtype. Notably, our data indicate that *RNF8* was significantly expressed in the BL1 and M types of TNBC (Figure 2). These data suggest that focusing on *RNF8* may address the detailed mechanism of DNA damage response and cell motility when acquiring the malignant phenotype of TNBC and further identify a personalized strategy for the development of anti-cancer agents.

In TNBC, conventional systemic CT is the mainstay of treatment. RT combined with CT was explored in LN+ patients. In addition, *BRCA1/2* germline mutation can be checked, and PARP inhibitors may be used as the first priority to treat the positive group [37]. However, there is an unmet need for a predictive biomarker for establishing a better therapy. In the present study, we identified that the *RNF8^high^*/*CDH1^low^* index not only represented a poor diagnostic marker but also indicated a poor treatment response to CT treatment alone. The combination of CT and RT could effectively improve the survival rate in patients with an *RNF8^high^*/*CDH1^low^* index compared to CT treatment alone. The above evidence suggests that *RNF8* may be used as a guiding clue for clinical classification, and the precise treatment can be further clarified in concurrent chemoradiation (CCRT) selection to avoid over-treatment. It is worth noting that the retrospective nature of current study might limit the inclusion of all relevant prognostic variables (e.g., demographic area, education level). In addition, the multi-factor interaction effect, especially the marginal effects, was not comprehensively considered, and further application of a machine learning algorithm was required to estimate the high-dimensional interaction between gene expression. Despite the mentioned limitations, this study proposes a novel data-driven therapeutic effect estimation approach based on an open access, real-world database, which could offer quick access to identifying potential anti-cancer candidate genes and provide added value for integrated cancer databases.

## 5. Conclusions

In our study, we first focused on different genes and noticed that enrichment in *RNF8* may be the most representative, also indicating a relatively poor prognosis, especially in the TNBC group. In addition, we found that the RNF8–CDH1 co-expression could predict the outcome of breast cancer. *RNF8^high^*/*CDH1^low^* functions as a poor prognostic marker in LN+ TNBC patients. This suggests different strategies for treatment. As we know, adjuvant radiotherapy may not be indicated for patients with a low level of LN metastasis (or micrometastasis group). However, according to our study, adjuvant radiotherapy to the axillary area may be suggested in patients with *RNF8^high^*/*CDH1^low^* due to the high recurrence rate, although the lymph nodal number is lower.

## Figures and Tables

**Table 1:** Baseline characteristics of study population (*n* = 498).

Variables	All	LN (−)	LN (+)	*p*
Cases, row %	498	234 (47.0%)	264 (53.0%)	
Age (years), median (range)	56 (26–90)	57 (29–89)	56 (26–90)	0.233
Subtype				0.002
Basal (TNBC)	114 (22.9%)	68 (29.1%)	46 (17.4%)	
Her2	42 (8.4%)	19 (8.1%)	23 (8.7%)	
LumA	220 (44.2%)	105 (44.9%)	115 (43.6%)	
LumB	122 (24.5%)	42 (17.9%)	80 (30.3%)	
T staging (AJCC)				<0.001
T1	152 (30.5%)	90 (38.5%)	62 (23.5%)	
T2	305 (61.2%)	135 (57.7%)	170 (64.4%)	
T3	33 (6.6%)	8 (3.4%)	25 (9.5%)	
T4	8 (1.6%)	1 (0.4%)	7 (2.7%)	
N staging (AJCC)				<0.001
N0	234 (47.0%)	234 (100%)	0 (0%)	
N1	181 (36.3%)	0 (0%)	181 (68.6%)	
N2	58 (11.6%)	0 (0%)	58 (22.0%)	
N3	25 (5.0%)	0 (0%)	25 (9.5%)	
Pathological Stage				<0.001
Stage I	94 (18.9%)	90 (38%)	4 (1.5%)	
Stage II	304 (61.0%)	143 (61%)	161 (61%)	
Stage III	100 (20.1%)	1 (0.4%)	99 (38%)	
Radiation	321 (64.5%)	129 (55%)	192 (73%)	<0.001
Chemotherapy	390 (78.3%)	167 (71%)	223 (84%)	<0.001
Hormone therapy	306 (61.4%)	136 (58.1%)	170 (64.4%)	0.179
Target therapy	306 (61.4%)	136 (58.1%)	170 (64.4%)	0.179

*p*-Value was estimated using independent two-sample *t*-test, chi-square test, or Fisher’s exact test.

**Table 2:** Correlation between RNF8 and involved genes.

Genes	*r* ^a^	*p*
*CDKN1A*	0.001	0.991
*AKT2*	0.047	0.292
*TGFB2*	0.037	0.404
*PDGFRA*	−0.076	0.092
*CDH1*	−0.162	<0.001
*SNAI1*	0.185	<0.001
*PEBP1*	0.010	0.818
*NPHS1*	0.065	0.145
*CDH2*	0.032	0.471

^a^ Correlation coefficient. *p*-Value was estimated using Pearson’s correlation test.

## Data Availability

The data presented in this study are available in the article.
