# Correction: Kao et al. RNF8–CDH1 Co-Expression Predicts Clinical Benefit of Chemoradiotherapy in Triple-Negative Breast Cancer. *J. Pers. Med.* 2021, *11*, 655

**DOI:** 10.3390/jpm12030466

**Published:** 2022-03-15

**Authors:** Chieh-Ni Kao, Sin-Hua Moi, Ming-Feng Hou, Chi-Wen Luo, Fang-Ming Chen, Mei-Ren Pan

**Affiliations:** 1 Graduate Institute of Clinical Medicine, Kaohsiung Medical University, Kaohsiung 807, Taiwan; jennykao0320@gmail.com; 2 Division of Breast Oncology and Surgery, Department of Surgery, Kaohsiung Medical University Hospital, Kaohsiung 807, Taiwan; cwlo0623@gmail.com (C.-W.L.); fmc5464@gmail.com (F.-M.C.); 3 Center of Cancer Program Development, E-Da Cancer Hospital, I-Shou University, Kaohsiung 824, Taiwan; moi9009@gmail.com; 4 Drug Development and Value Creation Research Center, Kaohsiung Medical University, Kaohsiung 807, Taiwan

The authors would like to make corrections to a recently published paper [1]. The reason for the correction is that Figure 5 was misplaced with all TNBC data, not TNBC LN (+) data. In addition, typo errors and citation errors were shown in Sections 3.1 and 3.2, and Reference No. 6.

Original Figure 5:

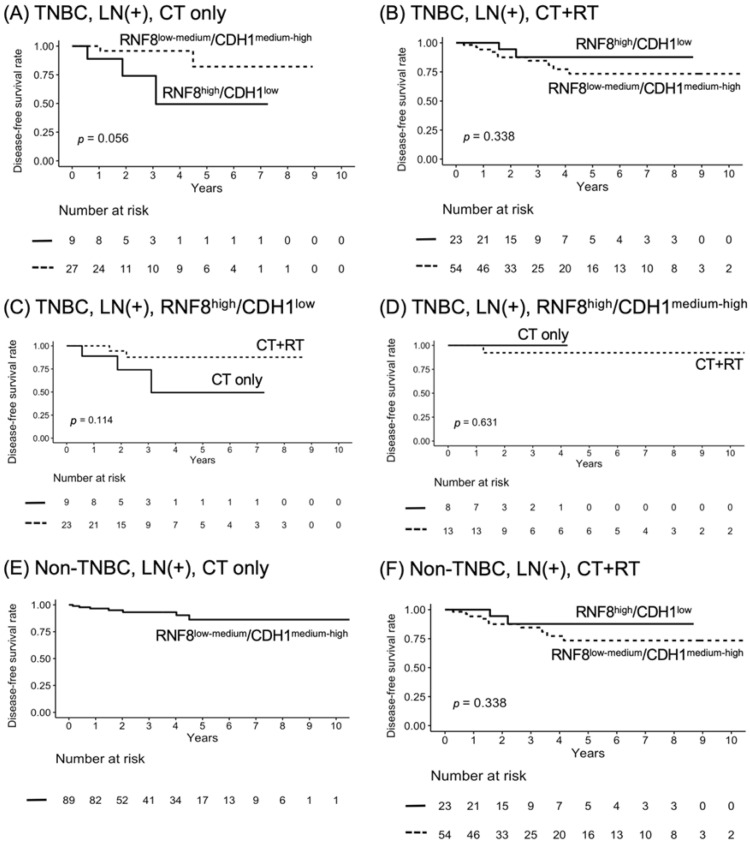

We would like it to be corrected as shown below.

1. New Figure 5:
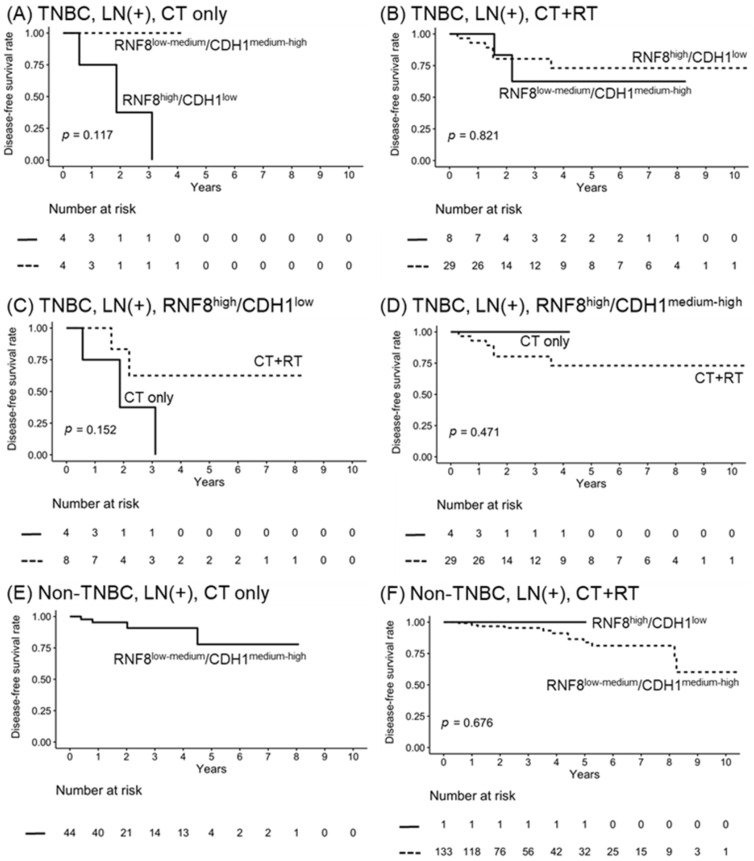

2. The result of Section 3.1. Clinicopathological Characteristics and Progression of Breast Cancer.

A total of 264 LN+ patients, with a median age of 56 years.

3. The result of Section 3.2. A Significant Correlation Among RNF8, SNAI1, and CDH1 mRNA Expression in Patients with Breast Cancer.

*SNAI1* showed a significant positive correlation (*r* value = 0.185).

4. The right reference No. 6 is: Kalluri, R.; Weinberg, R.A. The basics of epithelial-mesenchymal transition. *J. Clin. Investig.* **2009**, *119*, 1420–1428. http://doi.org/10.1172/jci39104.

These changes do not change the results or conclusions of our paper. The authors would like to apologize to readers of *JPM* for this error. The published version will be updated on the article webpage, with a reference to this correction notice.
